# Extending the clinical spectrum of X-linked Tonne-Kalscheuer syndrome (TOKAS): new insights from the fetal perspective

**DOI:** 10.1136/jmg-2024-109854

**Published:** 2024-06-07

**Authors:** Silvestre Cuinat, Chloé Quélin, Claire Effray, Christèle Dubourg, Gwenaelle Le Bouar, Anne-Sophie Cabaret-Dufour, Philippe Loget, Maia Proisy, Fanny Sauvestre, Mélie Sarreau, Sophie Martin-Berenguer, Claire Beneteau, Sophie Naudion, Vincent Michaud, Benoit Arveiler, Aurélien Trimouille, Pierre Macé, Sabine Sigaudy, Olga Glazunova, Julia Torrents, Laure Raymond, Marie-Hélène Saint-Frison, Tania Attié-Bitach, Mathilde Lefebvre, Yline Capri, Nicolas Bourgon, Christel Thauvin-Robinet, Frédéric Tran Mau-Them, Ange-Line Bruel, Antonio Vitobello, Anne-Sophie Denommé-Pichon, Laurence Faivre, Anne-Claire Brehin, Alice Goldenberg, Sophie Patrier-Sallebert, Alexandre Perani, Benjamin Dauriat, Sylvie Bourthoumieu, Catherine Yardin, Valentine Marquet, Marion Barnique, Maryse Fiorenza-Gasq, Isabelle Marey, Danielle Tournadre, Raïa Doumit, Frédérique Nugues, Tahsin Stefan Barakat, Francisco Bustos, Sylvie Jaillard, Erika Launay, Laurent Pasquier, Sylvie Odent

**Affiliations:** 1 Service de Génétique Clinique, CRMR anomalies du développement CLAD-Ouest, CHU Rennes, Rennes, France; 2 Service d'Anatomie et Cytologie Pathologiques, Hôpital Pontchaillou, CHU Rennes, Rennes, France; 3 Laboratoire de Génétique Moléculaire, Hôpital Pontchaillou, CHU Rennes, Rennes, France; 4 CNRS, INSERM UMR 6290, ERL U1305, F-35000, Université de Rennes, IGDR, Rennes, France; 5 Unité de Médecine fœtale, Service de Gynécologie-Obstétrique, CHU Rennes, Rennes, France; 6 Radiology Department, CHU de Brest, Brest, France; 7 Unité de Pathologie Fœto-placentaire, Service de Pathologie, CHU de Bordeaux, Bordeaux, France; 8 Department of Gynaecology and Obstetrics, Mother and Children's Hospital, CHU Limoges, Limoges, France; 9 Service de Génétique Médicale, CHU de Bordeaux, Bordeaux, France; 10 INSERM U1211, Maladies Rares, Génétique et Métabolisme, Université de Bordeaux, Bordeaux, France; 11 Institut méditerranéen d'imagerie médicale appliquée à la gynécologie, la grossesse et l'enfance IMAGE2, Marseille, France; 12 Département de Génétique Médicale, Hôpital Timone Enfant, AP-HM, Marseille, France; 13 Department of Pathology and Neuropathology, La Timone Hospital, Aix Marseille University, AP-HM, Marseille, France; 14 Genetics Department, Laboratoire Eurofins Biomnis, Lyon, France; 15 Foetopathology Unit, AP-HP Nord, Hôpital Robert Debré, Paris, France; 16 Service de Médecine Génomique des Maladies Rares, Hopital Universitaire Necker-Enfants Malades, AP-HP, Paris, France; 17 INSERM UMR 1163, Imagine Institute, Université Paris Cité, Paris, France; 18 Service de Pathologie fœtale, Hôpital Universitaire Armand Trousseau, AP-HP, Paris, France; 19 Département de Génétique, Hôpital Robert Debré, AP-HP, Paris, France; 20 Service d'Obstétrique-Maternité Chirurgie, Médecine et Imagerie foetales, AP-HP, Hopital Universitaire Necker-Enfants Malades, Paris, France; 21 UMR1231 GAD, INSERM, Université Bourgogne Franche-Comté, Dijon, France; 22 Unité Fonctionnelle Innovation en Diagnostic Génomique des Maladies Rares, FHU-TRANSLAD, CHU Dijon, Dijon, France; 23 Centre de référence Anomalies du Développement et Syndromes Malformatifs, Fédération Hospitalo-Universitaire TRANSLAD, CHU Dijon, Dijon, France; 24 Department of Pathology, Department of Genetics and Reference Center for Developmental Abnormalities, F-76000, CHU de Rouen, Rouen, France; 25 Inserm U1245, Université de Rouen Normandie, Rouen, France; 26 Department of Genetics and Reference Center for Developmental Abnormalities, F-76000, CHU de Rouen, Rouen, France; 27 Service de Fœtopathologie, CHU de Rouen, Rouen, France; 28 Cytogenetic, Medical Genetic and Reproductive Biology Department, Hôpital de la Mère et de l'Enfant, CHU Dupuytren, CHU Limoges, Limoges, France; 29 UMR 7252, CNRS, XLIM, F-87000, Université de Limoges, Limoges, France; 30 INSERM U1209, Institute for Advanced Bioscience, Université Grenoble Alpes, Grenoble, France; 31 CPDPN de Grenoble, Echographie obstétricale dépistage et diagnostic, CHU Grenoble Alpes, Grenoble, France; 32 Service d'Imagerie Pédiatrique, CHU Grenoble Alpes, Grenoble, France; 33 Department of Clinical Genetics, Erasmus MC University Medical Center, Rotterdam, The Netherlands; 34 ENCORE Expertise Center for Neurodevelopmental Disorders, Erasmus MC University Medical Center, Rotterdam, The Netherlands; 35 Discovery Unit, Department of Clinical Genetics, Erasmus MC University Medical Center, Rotterdam, The Netherlands; 36 Pediatrics and Rare Diseases Group, Sanford Research, Sioux Falls, South Dakota, USA; 37 Department of Pediatrics, Sanford School of Medicine, University of South Dakota, Vermillion, South Dakota, USA; 38 Service de Cytogénétique et Biologie Cellulaire, CHU Rennes, Rennes, France; 39 EHESP, INSERM U1085 IRSET, Université de Rennes 1, Rennes, France; 40 FHU GenoMeds, ERN ITHACA, CHU Rennes, Rennes, France

**Keywords:** Genetic Diseases, Inborn, Genetic Diseases, X-Linked, Genetics, Genomics, Exome Sequencing

## Abstract

**Introduction:**

Tonne-Kalscheuer syndrome (TOKAS) is a recessive X-linked multiple congenital anomaly disorder caused by *RLIM* variations. Of the 41 patients reported, only 7 antenatal cases were described.

**Method:**

After the antenatal diagnosis of TOKAS by exome analysis in a family followed for over 35 years because of multiple congenital anomalies in five male fetuses, a call for collaboration was made, resulting in a cohort of 11 previously unpublished cases.

**Results:**

We present a TOKAS antenatal cohort, describing 11 new cases in 6 French families. We report a high frequency of diaphragmatic hernia (9 of 11), differences in sex development (10 of 11) and various visceral malformations. We report some recurrent dysmorphic features, but also pontocerebellar hypoplasia, pre-auricular skin tags and olfactory bulb abnormalities previously unreported in the literature. Although no clear genotype–phenotype correlation has yet emerged, we show that a recurrent p.(Arg611Cys) variant accounts for 66% of fetal TOKAS cases. We also report two new likely pathogenic variants in *RLIM*, outside of the two previously known mutational hotspots.

**Conclusion:**

Overall, we present the first fetal cohort of TOKAS, describe the clinical features that made it a recognisable syndrome at fetopathological examination, and extend the phenotypical spectrum and the known genotype of this rare disorder.

**WHAT IS ALREADY KNOWN ON THIS TOPIC**

- Tonne-Kalscheuer syndrome (TOKAS) is a rare X-linked multiple congenital anomalies, which typically include intellectual disability, differences in sex development and congenital diaphragmatic hernia.

**WHAT THIS STUDY ADDS**

- We present 11 antenatal cases, describe novel symptoms and variants, and highlight a recurrent missense variant responsible for most fetal TOKAS cases in our series.

**HOW THIS STUDY MIGHT AFFECT RESEARCH, PRACTICE OR POLICY**

- This study will facilitate antenatal diagnosis of TOKAS and will enable these families to benefit from appropriate genetic counselling.

## Introduction

Tonne-Kalscheuer syndrome (TOKAS, MIM#300978) is an X-linked recessive multiple congenital anomaly disorder with two main presentations. Most male patients exhibit global developmental delay and cognitive impairment. Some more severely affected patients present with various congenital malformations. A recent cohort study showed that males with TOKAS present with intellectual disability (100% cases, mild to severe), visceral abnormalities including differences of sexual development (DSD, including micropenis, hypospadias or testicular hypoplasia; 90%), congenital diaphragmatic hernias (CDHs) (50%), congenital heart disease (17%), omphalocele (10%), cleft palate (8%), polysplenia (5%) and intestinal malrotation (5%). Other abnormalities include intrauterine growth restriction (IUGR; 80%), microcephaly (86%), short wide thumbs (88%), nail dysplasia (30%), camptodactyly (15%), syndactyly (10% on hands, 25% on feet) and pre-axial polydactyly (15%). Variable degrees of dysmorphic facial features are reported with prominent forehead, broad nasal root, malar hypoplasia and micrognathia. Of note, no cognitive impairment has been reported in carrier women, but premature ovarian failure and sometimes mild morphological features such as short broad thumbs are described.^1^

This syndrome has been associated with pathogenic variants in *RLIM* 2 on the X chromosome (Xq13.2), encoding for the RLIM (RNF12) ubiquitin ligase, a member of the ubiquitin-proteasome system (UPS). The UPS, main actor of the intracellular proteostasis, is a complex hub essential for neurodevelopment and embryogenesis.^3^ Ubiquitin chains are bound to intracellular proteins by various ubiquitin ligases to target them to the proteasome for degradation. About 1300 genes are involved in the UPS, mostly coding for ubiquitin ligases with specific substrates.^4^ Therefore, since the original reports associated *UBE3A* pathogenic variations with Angelman syndrome in 1997, 62 UPS genes have been linked with neurodevelopmental diseases.^5^

Within the UPS, RLIM ubiquitylates, many key factors are involved in essential cellular processes: REX1 (ZFP42) involved in X chromosome inactivation,^6^ oestrogen receptor-α involved in sexual determinism,^7^ TERF1 (TRF1) involved in telomere length,^8^ stathmin involved in cytoskeleton destabilisation,^9^ c-Myc and MDM2 involved in control of cell proliferation and differentiation,^10 11^ and SMAD7 which inhibits TGF-β and BMP signalling pathways playing an essential role in embryonic morphogenesis and cell migration.^12^ RLIM also influences neural stem cell differentiation^13 14^ and global transcriptional regulation recruiting the SIN3a/HDAC complex.^15^

To date, 9 pathogenic *RLIM* variants have been described in 41 patients.^1 2 16^ Only seven antenatal cases have been reported, often lacking phenotypical description. Here, we present the first TOKAS fetal cohort, describing 11 new cases in 6 French families.

## Material and methods

**Figure 1:**
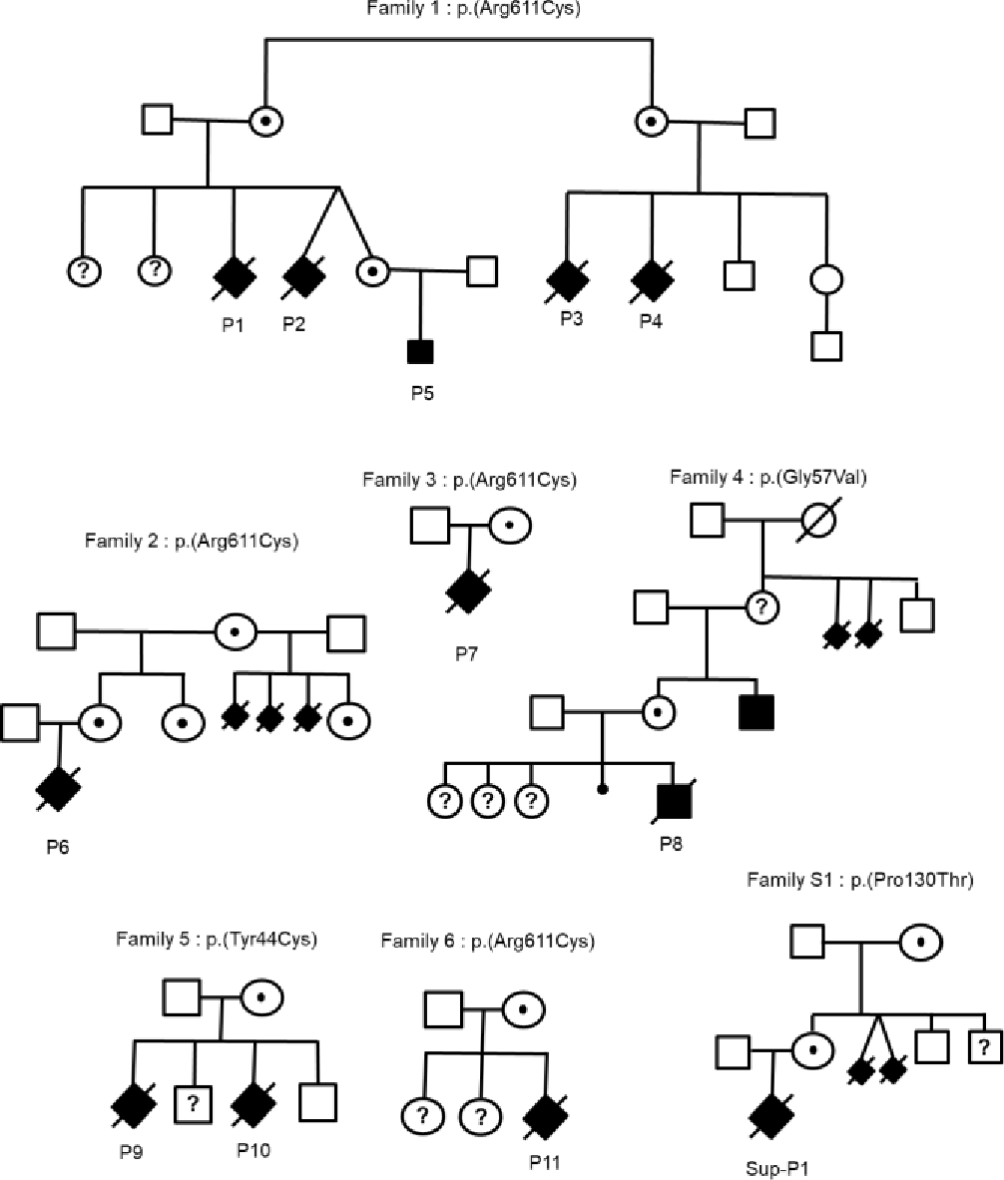
Family pedigree and corresponding variant of each antenatal Tonne-Kalscheuer syndrome case.

After a diagnosis of antenatal TOKAS made in a French family followed for over 35 years at Rennes University Medical Center (UMC), we launched a call for collaboration through the Société Française de Fœtopathologie, AnDDI-Rares (developmental abnormalities and malformative syndromes) and the European Reference Network on Rare Congenital Malformations and Rare Intellectual Disability. We collected clinical and molecular data on 12 fetuses from 7 unrelated families (pedigrees in figure 1) within 7 French UMCs. Among these fetuses, 9 of 12 were subject to autopsy, carried out by fetal pathologists, according to standardised protocols, including X-rays, photographs, macroscopic and histological examinations of all viscera. Fetal biometric data were evaluated using morphometric criteria of Guihard-Costa *et al* 17 in line with the fetopathological best practices. All families were evaluated by a clinical geneticist. Data on family history and antenatal imaging were provided in all cases. Informed consent for genetic testing was obtained from the couples. DNA was extracted from parental blood samples, amniotic fluid cells or fetal tissue. Trio exome sequencing (ES) was performed following routine protocols described in online supplemental data. All *RLIM* variants refer to the NM_016120.4 transcript (GRCh38/hg38). The search for a skewed X inactivation pattern was performed on blood sample from the mothers of families 5 and S1. For this, DNA was extracted and enzymatic digestion of the DNA with Hpall restriction enzyme was followed by PCR at the androgen receptor gene locus, including the CAG triplet repeat, following a previously published protocol.^18^

10.1136/jmg-2024-109854.supp1 Supplementary data

## Results

### Family 1

**Figure 2:**
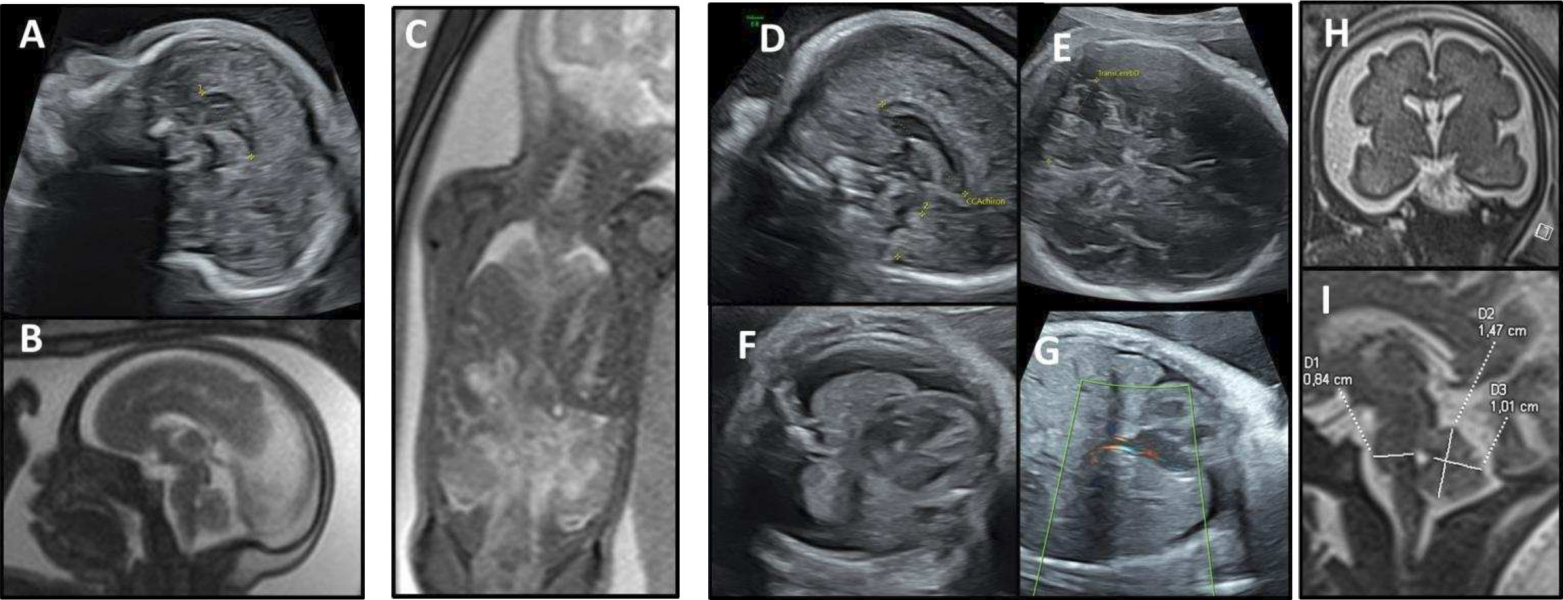
Prenatal imaging. Patient 5 (A) ultrasound at 24 GW then (B) MRI at 28 GW, both showing a short corpus callosum at the third percentile and the persistence of a nuchal thickening. Patient 6 (C) MRI at 29 GW, revealing a bilateral CDH, with intrathoracic right and left sides of the liver and an intrathoracic stomach. Patient 11 (D–I) showing at 27 GW ultrasound scans (D–G), a short corpus callosum at the third percentile (D), a cerebellar hypotrophy <1st percentile (E), a bilateral thoracic effusion (F) and a CDH with herniation of the right lobe of the liver (G). Fetal brain MRI at 30 GW (H,I) showing on coronal T2-weighted image a markedly enlarged subarachnoid space related to microcephaly (H), and on sagittal T2, biometries of the brainstem and cerebellum bellow the third percentile, supporting pontocerebellar hypoplasia (I). CDH, congenital diaphragmatic hernia; GW, gestational weeks.

The first family had been followed 35 years earlier for polymalformative syndrome in several male fetuses: the first couple, unrelated with no notable history, already had two healthy daughters. The third pregnancy was marked from 17 gestation weeks (GW) by a unilateral pleural effusion on fetal ultrasound (patient 1/P1). A female phenotype was observed on ultrasound at 23 GW, for a 46,XY fetal karyotype on amniotic fluid. At the couple’s request, medical termination of pregnancy (TOP) and autopsy were performed at 23+5 GW. Fetopathological examination (figure 2A) indicated that the fetus had normal biometry for the term. He showed abnormal facial features with hypertelorism, small and low-set ears, anteverted nostrils, long philtrum, gothic palate and retrognathism. He had on both hands single transverse palmar creases, large thumbs, brachytelephalangism on the second fingers and clinodactyly of the fifth fingers. At the foot, he had wide halluces, unilateral right polydactyly with toe buds in the first interdigital space, bilateral two to three cutaneous syndactyly, digital overlaps and hypoplasia of the first phalanx on all toes. DSD were confirmed with hypospadias, micropenis and hypoplastic testes. There was a horseshoe-shaped kidney, a retro-oesophageal right subclavian artery and an intestinal malrotation with complete common mesentery. The right pleural effusion was associated with hypoplasia of the ipsilateral lung, with no visualised diaphragmatic hernia.

During the fourth pregnancy, dichorionic diamniotic, a cystic hygroma with IUGR from 12 GW was observed in one twin (P2) with male-looking genital bud. At 18 GW, this fetus finally showed a female phenotype on the ultrasound scan, suggestive of DSD. Retrognathism with post-axial polydactyly and CDH were also observed. Karyotype on amniotic fluid revealed a 46,XY male genotype. Selective TOP of the symptomatic twin took place at 37 GW. He weighed 1910 g (0.15th percentile) and the autopsy was not requested by the parents (figure 2B). The second twin was a girl, 46,XX on karyotype, born healthy with normal biometry.

A similar history has been previously reported in the maternal aunt of P1 and P2, who had two pregnancies with comparable fetal symptoms. The first fetus (P3) presented IUGR at 24 GW. Hydramnios was observed at 32 GW. Premature delivery due to placental malperfusion at 32 GW led to neonatal death at 1 hour of life. Upon autopsy, the child weighed 1330 g (10th percentile). He showed abnormal facial features with hypertelorism, retrognathism and a gothic palate. He had a bilateral single transverse palmar crease, and his feet showed cutaneous syndactyly of the second, third and fourth toes. A bilateral CDH was observed. He had DSD with a feminine appearance of external genitalia, but no uterus, a rudimentary vagina and male gonads, with a 46,XY karyotype. During the second pregnancy of this couple, a left diaphragmatic hernia was suspected then confirmed, at 19 and 32 GW ultrasound, respectively. TOP and fetal autopsy were performed at 32 GW. The fetus (P4) showed dysmorphic features with low-set ears, a left CDH, DSD with hypoplastic penis and testes, rudimentary vagina and gonadal dysgenesis, for a 46,XY karyotype on amniotic fluid. The couple gave birth to a healthy boy and girl after the third and fourth pregnancies.

No molecular diagnosis could be made in this family until recently. 35 years after the first contact with the medical genetics department, the twin sister of patient 2, now adult at 8 GW, requested genetic counselling on her family history. Fetal sex determination by non-invasive prenatal screening was positive for Y chromosome material. The first ultrasound scans at 12 and 18 GW were normal, then the ultrasound at 22 GW showed a short corpus callosum, confirmed <3rd percentile by fetal MRI at 28 GW (figure 2A and B). No genital, diaphragmatic nor visceral anomalies were observed. Array-CGH was normal with a 46,XY karyotype. ES found a missense variant in *RLIM* (NM_016120.4:c.1831C>T p.(Arg611Cys)), predicted pathogenic with a Combined Annotation Dependent Depletion (CADD V.1.6) score of 32, absent from the control database (gnomAD V.4.0.0) and involving a highly conserved residue between species, with a Genomic Evolutionary Rate Profiling (GERP) score of 5.4. This variant was previously reported and demonstrated pathogenic, reducing the RLIM ubiquitin ligase activity.^1^ Thus, this variant established the diagnosis of TOKAS in this family. The variant could not be investigated in the four previous fetuses (P1–P4) due to the age of the samples. However, family segregation was consistent, and the variant found as expected in the mother, the maternal grandmother and the maternal great aunt, both asymptomatic. The couple did not opt for TOP. The child (P5) was born at full term with normal measurements and good adaptation to extrauterine life. Unilateral cryptorchidism with hypospadias and bilateral cutaneous syndactylies on toes were discovered. Abdominal ultrasound was normal, but cardiac ultrasound revealed ventricular septal defects and bicuspid aortic valve. Whole-body MRI confirmed the absence of diaphragmatic hernia but showed agenesis of the left olfactory bulb and hypoplasia of the right one.

### Family 2

**Figure 3:**
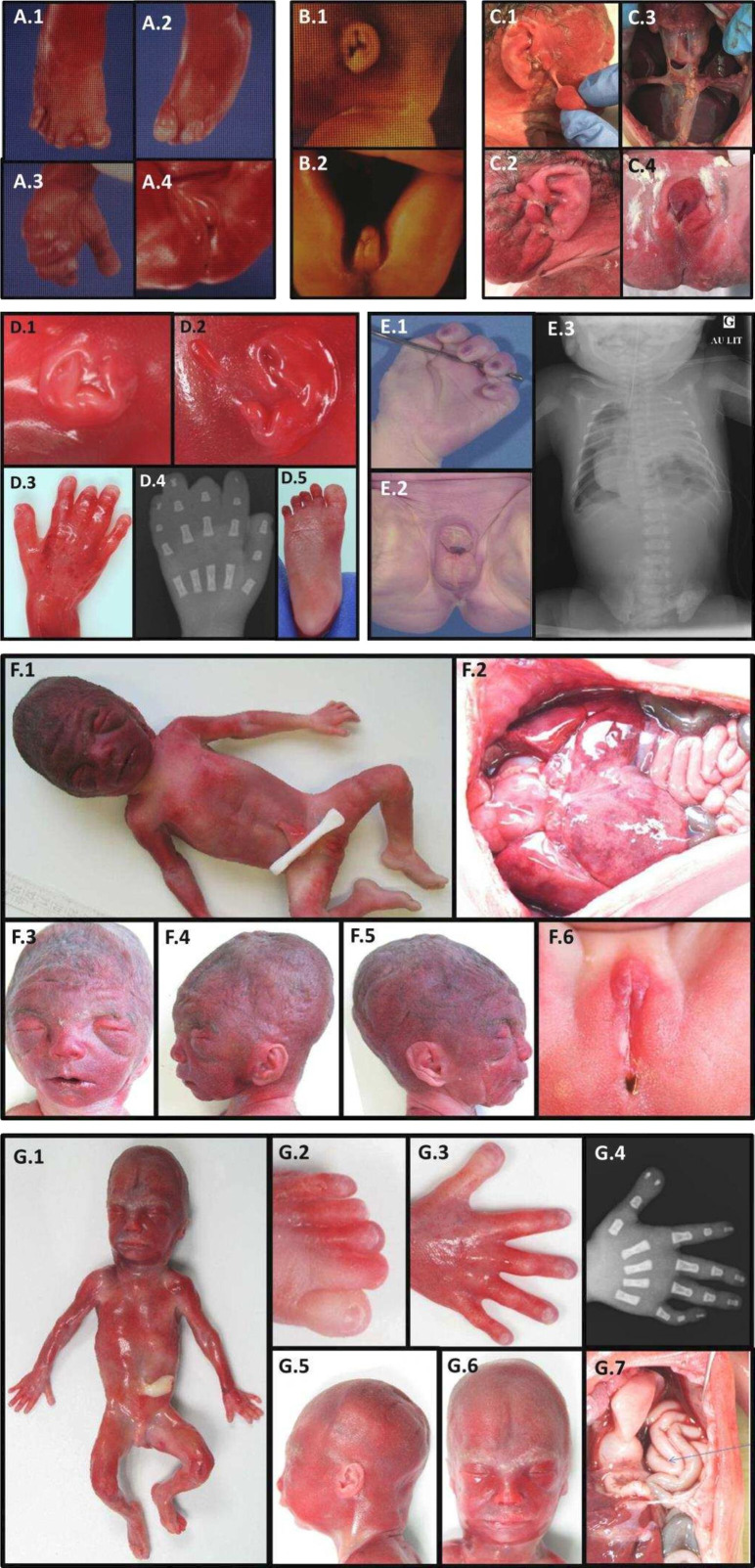
Postmortem findings. Patient 1 (A.1–A.4) at 23+5 GW, showing pre-axial polydactyly of the right foot (A.1), overlapping toes of the left foot (A.2), large thumb (A.3) and DSD (A.4). Patient 2 (B.1–B.2) at 37 GW showing small low-set ears (B1) and DSD (B.2). Patient 6 (C.1–C.4) at 31 GW. Note the large bilateral pre-auricular skin tags (C.1, C.2), bilateral posterior CDH with intrathoracic left and right sides of the liver (C.3), DSD with hypospadias and empty scrotum (C.4). Patient 7 (D.1–D.5) at 20 GW. Note a small right ear (D.1), two left pre-auricular skin tags (D.2), broad thumbs, short distal phalanges of the second and third fingers (D.3, D.4), broad halluces and short distal phalanx of the third toes (D.5). Patient 8 (E.1–E.3) at 38 GW, showing single transverse palmar crease (E.1), DSD with hypospadias (E.2), left CDH with ipsilateral lung hypoplasia and intrathoracic digestive tract on X-rays (E.3). Patient 9 (F.1–F.6) at 25 GW. Note abnormal facial shape with hypertelorism, anteverted nares, low-set ears and retrognathism (F.1, F.3–F.5), CDH (F.2) and DSD (F.6). Patient 10 (G.1–G.7) at 21 GW, showing DSD (G.1), microretrognathism (G.5–G.6), broad hallux and two to three-toe syndactyly (G.2), duplicate right thumb (G.3, G.4) and left CDH with intrathoracic digestive loops (G.7).

A polymalformative syndrome was observed in the first fetus (P6) of a healthy unrelated couple, whose maternal grandmother had had three stillborn babies with CDH from a previous marriage. Following a normal first-trimester ultrasound, a bilateral CDH was observed at 23 GW, then large bilateral pre-auricular skin tags at 28 GW ultrasound. These anomalies were confirmed on MRI at 29 GW, with intrathoracic right and left sides of the liver and an intrathoracic stomach (figure 3C). The array-CGH on amniotic fluid was normal, with 46,XY karyotype. The pregnancy was terminated at 31 GW. Upon autopsy (figure 2C), macroscopic examination revealed facial dysmorphia with a square face, large bilateral pre-auricular skin tags, proptosis, a bulbous nasal tip with anteverted nostrils and a thin upper lip. The fetus also showed hypoplasia of the fingernails and toenails. DSD was observed with micropenis, hypospadias and cryptorchidism. Biometrics for gestational age were normal. Study of the viscera revealed a CDH resulting in pulmonary hypoplasia with abnormal lobulation. There was also a Meckel’s diverticulum, a malformative cardiopathy with atrial and ventricular septal defects and left pyelectasis. Histological examination revealed ischaemic necrosis of the left intra-abdominal testis with focal fibrosclerosis but no dysplasia. ES revealed the same *RLIM* p.(Arg611Cys) missense variant, inherited from the mother, the maternal grandmother, aunt and female cousin, all asymptomatic. Genetic testing for this variant was not possible in the other three fetuses with CDH. The couple is currently undergoing pre-implantation diagnosis.

### Family 3

The first fetus (P7) of an unrelated couple with no relevant family history, obtained with In-Vitro Fertilization/ Intra-Cytoplasmic Sperm Injection (IVF/ICSI) technique, presented with increased nuchal translucency (5.1 mm), cystic hygroma and a crown-rump length below the fifth percentile at the first-trimester ultrasound. The ultrasound scan at 16 GW showed the persistence of an increased nuchal fold (5.3 mm) and early severe IUGR. Both karyotype and array-CGH in chorionic villi sampling at 13 GW were normal, 46,XY. Parents opted for medical TOP at 20 GW. The autopsy (figure 2D) showed a severe proportionate IUGR was confirmed, with the weight, the craniocaudal length and the head circumference below the fifth percentile, corresponding to a 3-week delay. The fetus presented with an abnormal facial shape, slight retrognathia, small low-set ears with two left pre-auricular skin tags. Skeletal abnormalities included broad thumbs and halluces, short distal phalanges of the second and third fingers, short distal phalanx of the third toes, and partial syndactyly of the second and third toes. Examination of the viscera showed bilateral posterior CDH with partial ascension of the left and right liver lobes into the thorax, severe lung hypoplasia, polysplenia and congenital heart defects with an aberrant right subclavian artery and a bicuspid aortic valve. External and internal genitalia were normal, including histological examination of the gonads. Histological examination revealed hepatic fibrosis with excessive bile duct ectasia and rare cystic renal collecting ducts. ES identified the same *RLIM* missense variation p.(Arg611Cys), inherited from the asymptomatic mother.

### Family 4

The patient (P8) was a male fetus from a spontaneous pregnancy in healthy, non-consanguineous parents. According to family history, a maternal uncle presented with intellectual disability, renal malformation, inguinal hernia, broad and short phalanxes of the second fingers, broad halluces and complete syndactyly of the second and third toes (online supplemental figure 1). The maternal grandmother had two stillborn babies with polymalformative syndrome. The couple already had a healthy daughter and had two early spontaneous miscarriages. The first-trimester ultrasound showed no abnormalities. The ultrasound scan at 22 GW revealed a left CDH with stomach, bowel and right kidney in the left hemithorax. The fetus showed moderate IUGR. Karyotype and array-CGH were normal, 46,XY, on amniotic fluid. A caesarean section was performed at 38 GW with urgent delivery due to fetal heart rate deceleration. The newborn died 8 hours after birth due to refractory hypoxia and severe lung hypoplasia. The autopsy (figure 2E) confirmed IUGR with a weight at the third percentile, a craniocaudal length at 0.5th percentile, but a normal head circumference (17th percentile). No skeletal abnormalities were reported. Abnormal facial features included a broad squared forehead, hypertelorism and thin lips. The fetus presented with DSD with hypospadias and undescended testes in the abdominal cavity. Examination of the viscera showed omphalomesenteric duct remnant, bilateral posterolateral CDH with stomach, spleen and bowel in left hemithorax, right kidney in the right hemithorax, severe bilateral lung hypoplasia and dextroposition of the heart. Histological examination did not reveal any significant abnormalities. ES identified a new *RLIM* missense variant NM_016120.4:c.170G>T p.(Gly57Val), inherited from the healthy mother, predicted pathogenic with a CADD V.1.6 score of 33, absent from the control database (gnomAD V.4.0.0) and involving a highly conserved residue between species, with a GERP score of 5.86. This variant was found in the asymptomatic maternal uncle and is considered explanatory.

10.1136/jmg-2024-109854.supp2 Supplementary data

### Family 5

A healthy unrelated couple with no significant family history had a first fetus (P9) with similar symptomatology: after a normal first-trimester ultrasound, the second-trimester ultrasound revealed harmonious IUGR with bilateral CDH, right renal agenesis and DSD with external genitalia of feminine appearance, with 46,XY karyotype on amniotic fluid. TOP was performed at 25 GW. Autopsy (figure 2F) revealed facial dysmorphism with hypertelorism, synophrys, low posteriorly rotated ears, anteverted nostrils, long philtrum and slight retrognathism. Biometrics were harmonious, at the fifth percentile. Visceral study confirmed bilateral wide CDH with bilateral pulmonary hypoplasia and polysplenia. Internal genitalia were undifferentiated with gonads in the iliac fossa, without uterus nor vagina. The couple subsequently had a boy born healthy, but the third fetus (P10) also showed mild IUGR, left CDH and DSD with undifferentiated external genitalia on 18 GW ultrasound. Array-CGH was normal, with 46,XY karyotype on amniotic fluid. TOP was performed at 20 GW. Fetal autopsy (figure 2G) revealed abnormal facial features with coarse face, low posteriorly rotated ears, hypertelorism, flat nasal root, anteverted nostrils, smooth philtrum and retrognathism. The thumbs and halluces were large, with duplication of the distal phalanx of the right thumb, bilateral cutaneous syndactyly of the second and third toes. Biometrics were normal for the term. The fetus also presented 13 pairs of ribs, a micropenis with hypospadias and male gonads in the iliac fossa. Visceral examination confirmed left CDH with bilateral pulmonary hypoplasia. Examination of the kidneys, heart and central nervous system (CNS) was normal. A new missense variant in *RLIM* NM_016120.4:c.131A>G p.(Tyr44Cys) was identified in these two fetuses, inherited from the asymptomatic mother. It was predicted pathogenic with a CADD score of 24.4, absent from gnomAD V.4.0.0 database and impacting a highly conserved residue with a GERP score of 5.47. As previously published for carrier women,^1^ a strong X inactivation skewing (99/1) was demonstrated in the mother, reinforcing the pathogenicity hypothesis for this variant. During the following pregnancy, a prenatal diagnosis was performed on trophoblast biopsy, giving birth to a healthy boy who does not carry the variant.

### Family 6

This unrelated couple with no significant family history already had two healthy daughters. During the third pregnancy, the fetus (P11) showed increased nuchal translucency of 3.8 mm and a short femur on first-trimester ultrasound. Amniocentesis was performed. The search for a cytomegalovirus infection was negative. Array-CGH was normal with 46,XY karyotype. At 27 GW, ultrasound showed normal biometry for term, but bilateral pleural effusion, with cerebellar hypotrophy <1st percentile and a corpus callosum at the third percentile (figure 3D–G). At 29 GW, ultrasound also revealed a left superior vena cava. Fetal MRI at 30 GW (figure 3H, I) also revealed a diaphragmatic hernia with hernia of right and left hepatic lobes, stomach, spleen and small bowel into the thoracic cavity. Pleural effusion was confirmed, with bilateral pulmonary hypoplasia. The fetal brain MRI showed markedly enlarged subarachnoid space related to microcephaly, confirmed cerebellar hypotrophy and also revealed an anteroposterior diameter of brainstem below third percentile. Antenatal ES revealed the recurrent *RLIM* missense variant p.(Arg611Cys), inherited from the asymptomatic mother. A medical TOP was requested by the couple, who did not request a fetal autopsy.

## Discussion

### Clinical characteristics in fetus with antenatal TOKAS

Since the first description of the syndrome, seven cases of antenatal TOKAS have been reported.^1 2 16^ Here, we report 11 new cases in 6 unrelated families. As expected, the summary of the 18 cases shows a high frequency of CDH and DSD (summarised in table 1; additional details given in online supplemental table 1), making these symptoms the main hallmarks in antenatal TOKAS. Facial dysmorphia appears variable and may not be recognisable, but includes some recurrent features, such as a square-shaped face, hypertelorism, small low-set ears, anteverted nostrils and a thin upper lip. Interestingly, pre-auricular skin tags, cerebellar, brainstem and olfactory bulb hypoplasia had not yet been reported. Limb anomalies are inconstant but may be suggestive, with brachytelephalangia of the third fingers and toes, large thumbs and halluces, syndactyly of the second and third toes, post-axial polydactyly and nail hypoplasia. Of note, morphological abnormalities of the CNS were among the first ultrasonographic signs in two fetuses (P5 and P11). These symptoms are unusual in TOKAS, and of the eight adult patients with brain MRI performed and reported in the literature, only one exhibited a corpus callosum anomaly,^1^ which also extends the phenotype.

**Table 1:** Summary of clinical characteristics in patients with antenatal TOKAS

Symptom	Prevalence
First antenatal sign	12–36 GW
CDH	89% (16/18)
Abnormal facial gestalt	72% (13/18)
DSD	72% (13/18)
Hand anomalies	44% (8/18)
Nail hypoplasia	28% (5/18)
Broad thumbs	17% (3/18)
Brachytelephalangia	17% (3/18)
Single palmar crease	11% (2/18)
Feet anomalies	50% (9/18)
Syndactylies	44% (8/19)
Nail hypoplasia	17% (3/18)
Broad halluces	17% (3/18)
Polydactylies	11% (2/18)
Microcephaly	40% (4/10)
IUGR	39% (7/18)
Digestive malformations	33% (6/18)
Nuchal thickening	22% (4/18)
Heart malformations	22% (4/18)
Large vessel malformations	17% (3/18)
Renal abnormalities	17% (3/18)
Hydramnios	11% (2/18)
CNS malformations	11% (2/18)
Pre-auricular skin tags	11% (2/18)
Hypo/aplasia of the olfactory bulbs	6% (1/18)

CDH, congenital diaphragmatic hernia; CNS, central nervous system; DSD, differences of sexual development; GW, gestational weeks; IUGR, intrauterine growth restriction; TOKAS, Tonne-Kalscheuer syndrome.

### Differential diagnoses

Different potential diagnoses were considered before ES allowed to make the diagnosis: syndromic CDH with genital abnormalities raised the hypothesis of Fryns syndrome (MIM#229850), but in this disease, there is an important prevalence of cleft palate, which is not reported in TOKAS. Donnai-Barrow (MIM#222448), Matthew-Wood (MIM#601186), microphthalmia, dermal aplasia, sclerocornea (MIM#309801) or microphthalmia type 12 (MIM#615524) syndromes also present with CDH, but are associated with ocular anomalies not reported in TOKAS. The association of DSD with IUGR and syndactyly 2–3 on the feet mainly evoked the Smith-Lemli-Opitz (MIM#270400) or Wolf-Hirschhorn (MIM#194190) syndromes, but CDH is rare in these diseases. Finally, when the case was not sporadic, none of these differential diagnoses could explain the family history, strongly suggestive of an X-linked recessive disease.

### *RLIM* pathogenic variants

**Figure 4:**
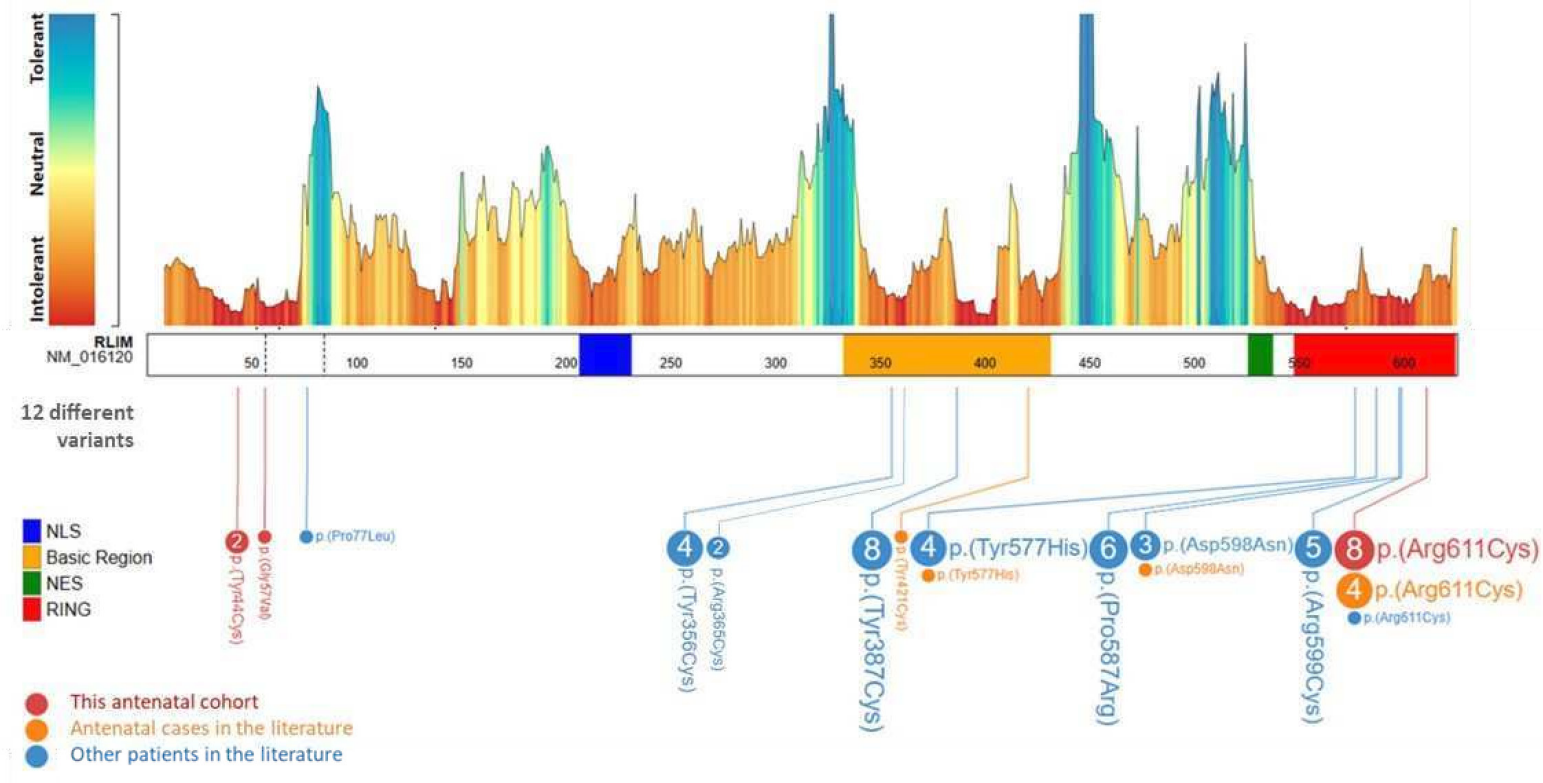
Genetic findings of individuals with *RLIM* variants. A schematic view of *RLIM* exon sequence (NM_16120.3), its *Metadome* missense tolerance landscape (from Radboud University Medical Center) and the variants identified in the different studies: new cases from our cohort in *red*, antenatal cases from literature in *orange* and postnatal cases from literature^1 2 16^ in *blue*. The amino acid numbering is based on PeCan Data of Proteinpaint. The domain representation is based on previous work by Gontan *et al* 6: nuclear localisation sequence (NLS) in *blue* (amino acid 207–231), basic region in *orange* (amino acid 333–431), nuclear export sequence (NES) in *green* (amino acid 526–537) and RING ubiquitin ligase domain in *red* (amino acid 548–624).

A total of 10 pathogenic missense variants in *RLIM* have already been reported in the literature (figure 4).^1 2 16^ Four of them were associated with antenatal TOKAS in four families previously reported.^1 16^ In the seven new families we present here, the same p.(Arg611Cys) variant was found in four families, making it the main provider of antenatal TOKAS and accounting for 66% of all cases (12 of 18). All these variants are inherited from asymptomatic mother. We therefore cannot exclude a founder effect that could explain the recurrence of the p.(Arg611Cys) variant. However, the lethality of this specific variant, and more generally the genotype–phenotype correlation in this syndrome, has not yet been explained. Of the 13 individuals carrying this variant, 12 had either an antenatal form with TOP or perinatal lethality. To date, the only individual who survived had a CDH with complex cardiac malformation, omphalocele and severe intellectual disability.^1^

No truncating variants have been reported and *RLIM* is predicted to be highly intolerant to loss-of-function variants according to gnomAD V.2.1.1 (pLI=1; o/e=0.05 (0.02–0.25)). This suggests early lethality of *RLIM* knockout in human, although *Rlim*−/Y knockout male mice are paradoxically viable.^19^ However, the fact that X chromosome microduplication involving *RLIM* also appears to be involved in a neurodevelopmental disorder in males suggests a strong dosage sensitivity of this gene for human neurodevelopment.^20^

Most described *RLIM* variants are located in two known mutational hotspots,^1^ namely the catalytic (RING) domain, responsible for ubiquitin ligase activity, and the basic region, required for self-association and auto-ubiquitylation, substrate and deubiquitylase binding.^6 21^ The p.(Arg611Cys) variant affects the RING domain and alters ubiquitin ligase activity, similarly to four other previously described variants.^1^ In particular, the Arg611 residue of RLIM corresponds to the linchpin arginine.^22^ This is a conserved residue in RING E3 ligases that mediates both E2 and ubiquitin interaction.^23^ The severe phenotype associated with this specific variant could be associated with disruption of these interactions. Furthermore, most variants described here introduce a cysteine residue into the protein sequence. Mutation to cysteine is classified as one of the most disease causing among other amino acid changes.^24^ This is likely due to the multiple functions of cysteine in mediating disulfide bonds, metal ion coordination and redox sensing, among others.^25^ Thus, introduction of cysteine residues by gene variants can promote neomorphic pathogenic functions. However, more studies are required to confirm the exact mechanisms by which these variants affect RLIM protein biology and drive disease.

We report here two undescribed *RLIM* variants within the *RLIM* amino-terminal region, located outside the two mutational hotspots but predicted highly intolerant to missense variations (figure 4). The potential impact of these p.(Gly57Val) and p.(Tyr44Cys) variants on RLIM ubiquitin ligase activity is uncertain. However, an autoinhibitory mechanism was recently reported, whereby the *RLIM* amino-terminus suppresses the basic region-mediated RLIM chromatin recruitment and substrate targeting.^26^ Further functional studies are required to understand the impact of these variants. Finally, it is noteworthy that the three patients in families 4 and 5, respectively, carriers of hemizygous p.(Gly57Val) and p.(Tyr44Cys) variants, showed a phenotype very consistent with TOKAS.^1^

A strong X inactivation skewing was demonstrated in the mother from family 5 with p.(Tyr44Cys), carrying the p.(Tyr44Cys) variant. This test has not been carried out on other mothers in our cohort, but Frints *et al* showed a similar pattern in 13 of 14 carrier mothers tested,^1^ suggesting that this test could support the pathogenicity of *RLIM* variant. An additional patient (online supplemental family S1, online supplemental data and online supplemental table 2), carrier of a new p.(Pro130Thr) variant, raises the question of a novel neurological phenotype. However, the absence of a significant X inactivation skewing pattern in the carrier mother currently suggests that it is not causal. Further investigations are required to prove its pathogenicity and exclude a different cause.

## Conclusion

Overall, we present here the first fetal cohort of TOKAS, and describe a suggestive pattern of visceral, skeletal and facial abnormalities, making it a recognisable syndrome upon fetopathological examination. We demonstrate the importance of considering this diagnosis in syndromic forms of CDH and DSD, and extend the phenotypical spectrum, reporting pre-auricular tags, olfactory bulb abnormalities, and cerebellar hypoplasia in some patients. At the molecular level, we show that a recurrent *RLIM* p.(Arg611Cys) variation is responsible for 66% of fetal forms of TOKAS, suggesting a genotype–phenotype correlation. We also report two new *RLIM* variants, located outside of the two previously known mutational hotspots, for which the pathomechanism will be investigated.

## Data Availability

All data relevant to the study are included in the article or uploaded as supplemental information.
