# Supplementary material for: Extending the clinical spectrum of X-linked Tonne-Kalscheuer syndrome (TOKAS): new insights from the fetal perspective
Source: J Med Genet. 2024 Jun 7;61(9):824–32. doi: 10.1136/jmg-2024-109854 (PMC11420740; doi:10.1136/jmg-2024-109854)
Supplement: Supplementary data [file jmg-2024-109854supp001.pdf]

SUPPLEMENTAL DATA

SUPPLEMENTAL MATERIAL AND METHODS

Patient 5 (Rennes)

Patient 5 was diagnosed by ES, following the protocol from Rennes UMC, after DNA extraction on cultured amniocytes. Sequencing technologies: Samples were prepared with the Agilent SureSelect Exome-All exons V8 preparation kit and sequenced on an Illumina NextSeq550 sequencer using 2x150 bp sequencing kits (Illumina, San Diego, California, USA). Briefly, gDNA was first fragmented by enzymes and DNA ends were modified with adaptor-tag for target enrichment. After amplification and purification, adaptor-ligated libraries were hybridized to SureSelect Exome and captured prior to indexing. Samples were then pooled for multiplexed sequencing. Bioinformatics pipeline: Raw sequencing data were mapped to the GRCh37/hg19 reference genome with the bwa-mem aligner (Li et al 2009). The identification of variants was performed using Freebayes and the GATK (Unified Genotyper and Haplotype Caller) (Van der Auwera 2013). When possible, the calling has been conducted in a family structure. Variants were annotated using ANNOVAR (Wang et al, 2010, build of February 2016) based on RefSeq genes, known variation from dbSNP144, and Clinvar and frequencies from the 1000 Genomes Project, esp6500 project, Exac (Lek et al 2016) and kaviar (Glusman et al. 2011) databases. The functional consequence of missense coding variants has been assessed using dbNSFP v.3.0 (Liu et al, 2015) that includes several deleteriousness prediction algorithms (SIFT, PolyPhen2 HDIV, PolyPhen2 HVAR, LRT, MutationTaster, MutationAssessor, FATHMM, MetaSVM, MetaLR, VEST, CADD, GERP++, DANN and PROVEAN). In order to facilitate the interpretation of these results, a summarizing value reflecting the percentage of predictions that classify this variant as deleterious has been calculated. For splices site prediction annotations we used the Annovar library dbscsnv1.1() that contain splices site prediction by AdaBoost and RandomForest. These annotations have been completed by custom made annotations: OMIM database / inheritance mode for each gene and the frequency of the variant within our exomic data. Variant have then be filtered based on the sequencing quality: a read-depth of ≥ 10× and a quality-score of ≥ 30 ; the type of variant : synonymous or non-coding variants other than those affecting splice-sites have been disregarded) and frequency in population : only variants with minor allele frequency <= 0.001 in any population database or our local database have been kept. For each sample, variants were investigated based on all likely transmission modes (autosomal-dominant, autosomal-recessive, X-linked-recessive in males). Segregation was used to filter further variants on family analysis. A list of X potential candidate genes for neurodevelopment disorders has been established from literature, OMIM, MGI database and Malacard web site. The variants were further prioritized based on the variant type, the deleteriousness predictions, OMIM and ClinVar information and the potential candidate gene list.

Patient 6 (Marseille)

For patient P6, DNA was extracted from fetal frozen tissues (lung) and parental peripheral whole-blood samples. Exome capture and sequencing were performed at Integragen (IntegraGen, Évry, France) from 1 µg of genomic DNA per individual using the SureSelect Human All Exon V5 kit

(Agilent). The resulting libraries were sequenced on a HiSeq 2000 (Illumina) according to the manufacturer's recommendations for paired-end 76 bp reads. Reads were aligned to the human genome reference sequence (GRCh37/hg19 build of UCSC Genome Browser) with the Burrows-Wheeler Aligner (BWA, v.0.6.2). The Genome Analysis Toolkit (GATK) v.2.6-4 was used for base quality score recalibration, indel realignment, and variant discovery (both single-nucleotide variants and indels). Variants were annotated with SeattleSeq SNP Annotation. Rare variants were identified by focusing on protein-altering and splice-site changes present at a frequency of less than 1% in dbSNP 138, Exome Aggregation Consortium (ExAC) and gnomAD. Total genomic coverage was better than ten reads for each rare variant. The clinical singleton ES analysis restricted to OMIM-related diseases gene was performed in two steps. First step used different strategies based on bioinformatics scores, variant and map databases for filtering variants and the second step was based on phenotype-genotype correlation. Variants were visualized on the Integrative Genomic Viewer before validation and familial segregation verified by Sanger sequencing.

Patient 7 (Paris/Dijon)

For patient 7, DNA was extracted from fetal lung and parental peripheral blood samples following standard procedures. Exome capture and sequencing were performed at IntegraGen (IntegraGen, Évry, France) from 1 µg of genomic DNA per individual using the TWIG kit. The resulting libraries were sequenced on an Illumina NovaSeq 6000 (Illumina, San Diego, California, USA) according to the manufacturer's recommendations for paired-end 101-bp reads. A mean depth of 110 x was reach and 97.5% of the RefSeq exons were covered at least by 10 reads.

Variants were identified using a computational platform of the FHU TRANSLAD, hosted by the University of Burgundy Computing Cluster (CCuB). Raw data quality was evaluated by FastQC software (v0.11.4). Reads were aligned to the human genome reference sequence (GRCh37 assembly) using BWA (Burrows-Wheeler Aligner; v0.7.15).

Aligned read data underwent the following steps: (a) duplicate paired-end reads were removed using Picard MarkDuplicates (version 2.4.1), and (b) base quality score recalibration was done by the Genome Analysis Toolkit (GATK v3.8) Base recalibrator. Using GATK Haplotype Caller, Single Nucleotide Variants with a quality score > 30 and an alignment quality score > 20 were annotated with SNPEff (v4.3). Rare variants were identified by focusing on nonsynonymous changes present at a frequency less than 1% in the gnomAD database. Copy number variants were detected using XHMM (v1.0) and were annotated using chromosomal coordinates of coding exonic sequences on the human genome (https://www.ncbi.nlm.nih.gov/refseq/) with in-house Python scripts. They were filtered regarding their frequency in public databases (DGV, ISCA, DDD). Mobidetails was used to help interpretation.

Variant confirmation and parental segregation were performed by Sanger sequencing using a routine diagnostic workflow. Primers were designed using the Primer3Plus web interface to the Primer3 program. Sequences were interpreted using FinchTV (v1.4.0) (Geospiza, Inc.; Seattle, WA, USA; http://www.geospiza.com).

Patient 8 (Rouen/Dijon)

Patient 8 was diagnosed by ES on DNA extracted from fetal muscular tissue, following the previously described protocol (**PMID: 32732226**), by the bioinformatic platform of FHU TRANSLAD (Université Dijon Bourgogne).

Patient 9 and 10 (Limoges)

For Patient 9 and 10, DNA was extracted from fetal muscular tissue. Exome sequencing followed the local diagnostic protocol. Library preparation, exome capture, sequencing and data analysis have been performed by IntegraGen (Integragen, Evry, France), using Twist Bioscience in-solution enrichment methodology (Agilent, Santa Clara, California), followed by paired-end 75 bases massively parallel sequencing on Illumina HiSeq4000 (Illumina, San Diego, California).

Patient 11 (Grenoble)

Patient 11 was diagnosed by ES on DNA extracted from fetal tissue, following the local protocol from Grenoble UMC for diagnostic. Fetal DNA was extracted with Maxwell (Promega) 16LEV Blood DNA kit. Parental DNA was extracted with Maxwell RSC (Promega) Bully Coat DNA kit or 16LEV Blood DNA kit. High-throughput exome sequencing was performed using Agilent Human All Exon v8 for libraries, then Illumina Paired-end 2x150pb on NextSeq500/550 (Illumina) for sequencing. Bioinformatics analysis were performed with SeqOneO v.1.2 software. Confirmation technique: Sanger sequencing of variants classified 4, 5 ACMG according to guidelines (PMID 29300386). Fetal samples were tested for maternal contamination using ldentifilero AmpFLSTR kit (ThermoFisher). Candidate variants met quality criteria: coverage > 30X: 99.58% (expected: >90%), average depth: 250.71X (expected: >30X).

Patient S1 (Bordeaux)

For Patient S1, DNA was extracted from fetal muscular tissue. Exome sequencing followed the local diagnostic protocol. Library preparation and exome capture have been done using SureSelect Human All Exon V8 kit (Agilent), followed by paired-end 75 bases massively parallel sequencing on Illumina NextSeq 550Dx (Illumina).

SUPPLEMENTAL RESULTS

Family S1: A more complex case is reported here, with the first male fetus (Sup-P1) of a healthy couple with no notable family history, who presented isolated CNS anomalies. On the 22 GW ultrasound, ventriculomegaly with periventricular nodular heterotopias (PVNH), asymmetric cerebellar hypotrophy and a short, thick corpus callosum were observed. The CMV research and the Array-CGH performed on amniotic fluid were normal, with a 46,XY karyotype. Medical abortion was

performed at 24 GW. At autopsy (**Supplemental Figure 2**.), the fetus showed discrete facial dysmorphia with low-set ears, anteverted nostrils, a thin upper lip, discrete retrognathism and clinodactyly of the fifth fingers on the hands. CNS abnormalities were confirmed, but no other visceral abnormalities were identified, notably no CDH nor DSD. Trio ES revealed a new *RLIM* missense variant NM_016120.4:c.388C>A p.(Pro130Thr), inherited from the asymptomatic mother, present in 1 woman in gnomAD v4.0.0 database, involving a highly conserved residue with a GERP score of 6.17 and predicted as pathogenic by in silico prediction scores including a CADD score of 25.7, but classified as variant of undetermined significance (VUS) due to clinical discordance with the expected phenotype. No other candidate variant has been identified. The family segregation study found this variant inherited from the asymptomatic mother and the grandmother, for whom the search for X chromosome inactivation bias was not significant (76/26 and 61/39, respectively). The variant was also investigated in a maternal uncle, who is not carrier.

SUPPLEMENTAL FIGURES

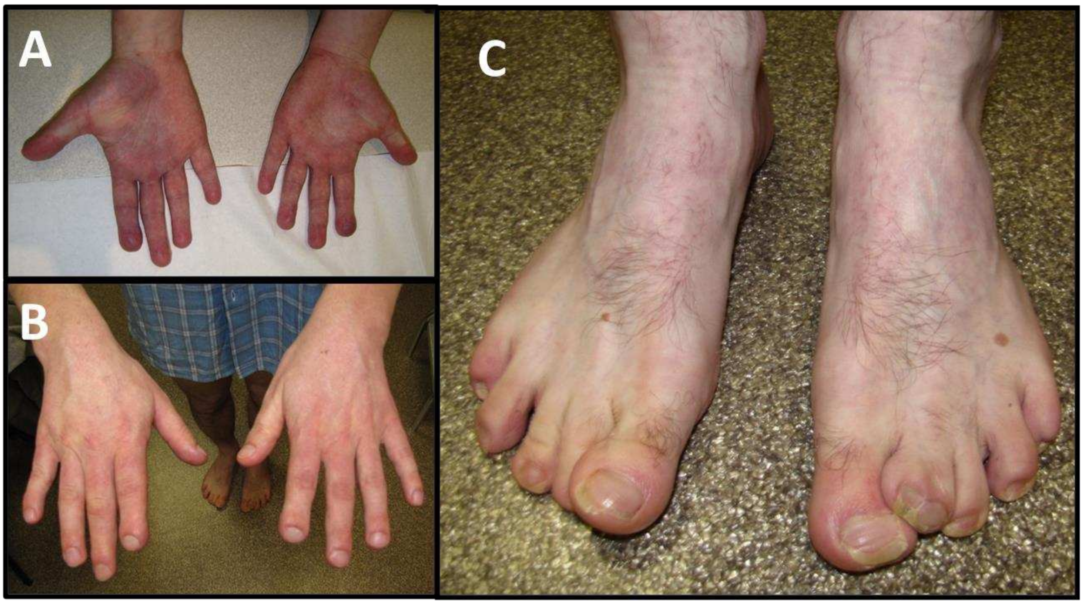

SUPPLEMENTAL FIGURE 1. Morphological features in the maternal uncle of P8 and P9 in family 4, carrier of the RLIM variant p.(Gly57Val). On the hands (A, B), note the bilateral brachytelephalangism of the second, third and fourth fingers with broad extremities. On the feet (C), note the broad halluces, the digital overlaps and the complete left 2-3 syndactyly.

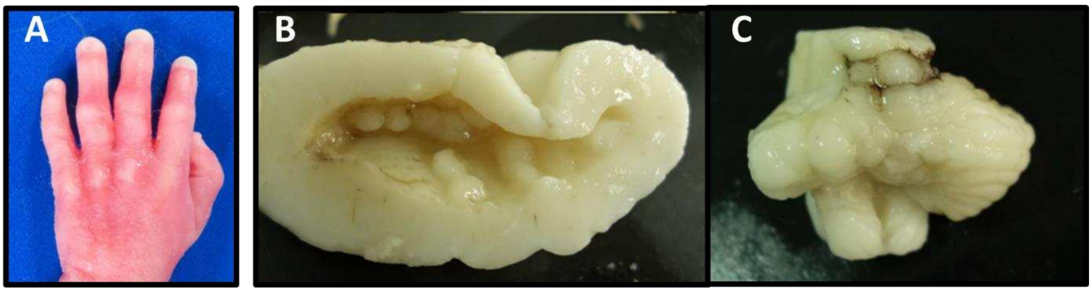

SUPPLEMENTAL FIGURE 2. Patient S1 (A – C) at 24 GW showing fifth fingers clinodactyly (A). At neuropathological examination, note periventricular nodular heterotopia with ventriculomegaly (B) and asymmetric cerebellar hypoplasia predominantly impacting the left hemisphere (C).
